# Reduction of Hexavalent Chromium from Soil of the Relocated Factory Area with Rice Straw Hydrothermal Carbon Modified by Nano Zero-Valent Iron (nZVI)

**DOI:** 10.3390/ijerph20043089

**Published:** 2023-02-10

**Authors:** Wei Zhong, Weiyang Bai, Gang Li

**Affiliations:** Department of Chemistry and Chemical Engineering, Chongqing University of Technology, Chongqing 400054, China

**Keywords:** rice straw, hydrothermal carbon, nano zero-valent iron, hexavalent chromium, adsorption kinetics

## Abstract

In order to reduce the content of Cr(VI) in the soil of the relocated chromium salt factory, the rice straw-derived hydrothermal carbon was prepared by hydrothermal method and loaded with nano zero-valent iron generated by liquid phase reduction, which effectively alleviated the self-aggregation problem of nano zero-valent iron (nZVI) in the treatment of Cr(VI) and improved the Cr(VI) reduction rate without changing the soil structure. The reduction effect of Cr(VI) in soil by key influencing factors such as carbon-iron ratio, initial pH value, and initial temperature was investigated. The results showed that nZVI modified hydro-thermal carbon composite (named RC-nZVI) had a good reduction effect on Cr(VI). Scanning electron microscope (SEM) and energy spectrum analysis showed that nZVI was evenly distributed on the surface of hydrothermal carbon, which effectively reduced the agglomeration of iron. Under the conditions of C/Fe = 1:2, 60 °C, with pH of 2, the average Cr(VI) content in soil decreased from 182.9 mg kg^−1^ to 21.6 mg kg^−1^. Adsorption kinetics of Cr(VI) by RC-nZVI fit well with the pseudo-second-order model, and the kinetic velocity constant revealed that Cr(VI) reduction rate decreased with increasing initial Cr(VI) concentration. Cr(VI) reduction by RC-nZVI was mainly dominated by chemical adsorption.

## 1. Introduction

China’s annual production of chrome salt and its series of chemical raw materials is more than 3,035,000 tons, widely used in tanning agents, metal plating, industrial pigments, and anti-corrosion and other modern processes, accounting for 10% of the national economy commodity varieties [1]. Chromium slag and chromium-containing industrial wastewater generated in the production process enter the environment without proper treatment, causing pollution to surface water and groundwater and damaging human health. Chromium primarily exists in the form of trivalent chromium Cr(III) and hexavalent chromium Cr(VI) in water [2]. Usually, the toxicity of Cr(VI) is two orders of magnitude higher than that of Cr(III) [3,4]. Cr(VI) mainly exists in the forms of CrO_4_^2−^, Cr_2_O_7_^2−^, and HCrO^4−^, which have strong migration and absorption, and have carcinogenicity after entering the human body [5]. Cr(VI) exists in the form of Cr(H_2_O)_6_^3+^, Cr(H_2_O)^3+^, and CrO^+^ in vitro, and more than 90% is adsorbed and fixed or formed precipitation, which is generally not toxic. Generally speaking, high-toxic Cr(VI) was reduced to non-toxic Cr(III) in environmental treatment [6].

With the continuous development of nanomaterials science, nanomaterials are widely used in environmental remediation because of their unique surface effect, volume effect, quantum size, and macroscopic quantum tunneling effect [7,8]. The nanoscale particle size of nano zero-valent iron (nZVI) gives it a large specific surface area (25–35 m^2^ g^−1^) and surface energy, thus exhibiting an extremely strong reducing and reacting activity. When it rapidly adsorbs Cr(VI) immobilized on the iron surface, the redox reaction between nZVI and Cr^6+^ reduces Cr(VI) to Cr(III), thus decreasing the Cr(VI) toxicity [5,9]. However, nZVI has functional limitations in practical field applications, with magnetic attraction between nano metallic particles, easy clustering of particles, and oxidation in air, which passivates and reduces the treatment capacity [10]. The results show that immobilizing nanoparticles on immobilized supports (activated carbon, metal oxides, bauxite, hydrothermal carbon) is an effective way to mitigate the nZVI agglomeration phenomenon and improve the stability of nZVI in the air [11,12]. Activated carbon is a widely used adsorbent material with a wide range of sources and low cost [13,14]. Activated carbon is generally prepared by biomass roasting method, which is prone to secondary air pollution such as particulate matter, CO, and NO_x_ during the preparation process [15,16].

In order to avoid air pollution in the process of activated carbon preparation, hydrothermal carbon (HTC) in the process of water preparation comes into view. Hydrothermal carbon (HTC) is a method for the conversion of lignocellulosic biomass into valuable carbon materials under a closed aqueous solution at a temperature of 100–300 °C and a pressure of 2–20 MPa (generally subcritical water) for more than 1 h [17,18]. It is a homogeneous, hydrophobic porous-structured solid containing micron or nano-sized carbon spheres [19]. In the high-pressure closed hydrothermal process, a large number of organic oxygen-containing functional groups are retained on the product’s surface, and the final product mainly contains amorphous carbon structure [20,21]. It has high-temperature resistance, radiation resistance, and good chemical stability in acid and alkali solutions, and different monomers can provide different functional groups, which provides excellent prerequisites for the adsorption and separation of heavy metal ions in aqueous solution [22]. KOH/NH_4_Cl was hydrothermal carbonized at 120–280 °C and 0.4–6.5 MPa, and the nitro-doped biochar was prepared by Shasha et al. [23]. The highest N content of biochar can reach 6.18%, and the central N species involved in graphite N, pyrrole N, and pyridine N. Nitrogen-doped biochar showed adequate adsorption capacity of Cu^2+^, Pb^2+^, Zn^2+^, and Cr^6+^. A decouple temperature and pressure hydrothermal (DTPH) reaction system was developed by Shijie et al. [18]. In this process, the HTC reaction of lignocellulosic biomass can be achieved at a temperature of 200 °C, breaking the temperature limit of the conventional process (230 °C). The produced hydrogen carbon is carbon microspheres, with high calorific value, rich oxygen-containing functional groups, high degree of graphitization, and good thermal stability.

China produces more than 500 million tons of grain every year, and rice produces more than 800 million tons of theoretical straw resources (687 million tons of collectable resources). Most of the straw is burned and stacked, which is harmful to the atmosphere [24]. Rice straw contains many celluloses, hemicellulose, and lignin. It has the advantages of improving soil structure and enhancing organic matter content and is one of the high-quality raw materials for preparing hydrothermal carbon [25,26]. However, the use of prepared rice straw high-performance hydrothermal carbon loaded with nZVI to the reduction of hexavalent chromium from contaminated soil has rarely been reported.

In this study, the rice straw hydrothermal carbon loaded nZVI particles were prepared by reducing method in liquid phase with hydrothermal carbon as carrier and FeSO_4_ and NaBH_4_ as raw materials. Results illustrated that different C/Fe ratio, pH, temperature, and initial Cr(VI) concentration have different effects on Cr(VI) reduction from contaminated soil at the former site of the chromium salt plant. The synergistic effects of hydrothermal carbon and nZVI were discussed, and the reduction mechanism was preliminarily studied to provide a theoretical foundation for large-scale application.

## 2. Materials and Methods

### 2.1. Materials and Chemicals

Ferrous sulfate (FeSO_4_·7H_2_O), potassium dichromate (K_2_Cr_2_O_7_), sodium borohydride (NaHB), and anhydrous alcohol (C_2_H_5_OH) were purchased from Sinopharm Chemical Reagent. Sulfuric acid (H_2_SO_4_) and phosphoric acid (H_3_PO_4_) were supplied by Tianjin Kemiou Chemical Reagent Co., Ltd., Tianjin, China. All adopted chemicals in this study were analytical-grade reagents. Standard soil samples: GBW07458 (ASA-7) organic matter (Institute of Geophysical and Geochemical Exploration, Chinese Academy of Geological Sciences).

### 2.2. Preparation of RC-nZVI

**Figure 1:**
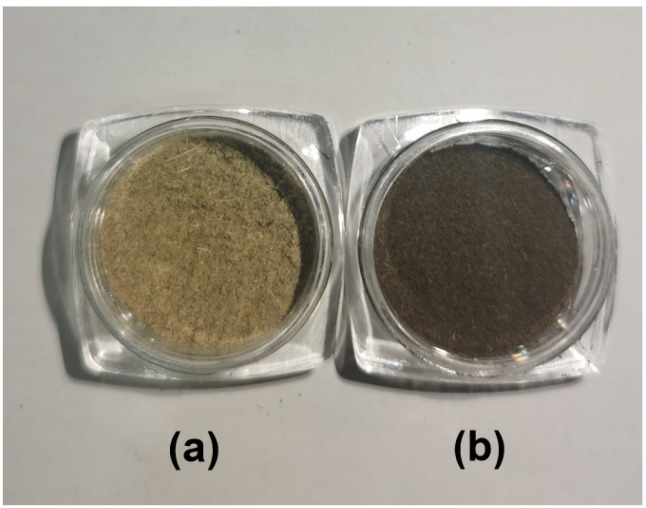
Materials prepared in the laboratory: (**a**) rice straw power; (**b**) rice straw hydrothermal carbon.

The rice straw was repeatedly washed with deionized water, dried at 80 °C, and crushed into 60 mesh sieves. An amount of 5 g of rice straw powder was weighed and placed in a hydrothermal reactor. According to the solid-liquid ratio of 1:8, 40 mL of distilled water was added in a stainless-steel reactor, stirred evenly. After 6 h of hydrothermal carbonization at 150 °C, the rice straw was cooled to the room temperature cup, washed with distilled water, filtered, and dried at 100 °C to obtain rice straw hydrothermal carbon (RC). All the prepared materials are shown in Figure 1.

A total of 5 g of FeSO_4_·7H_2_O and 5 g of hydrothermal carbon were weighed and dissolved in a 100 mL beaker with 100 mL MIlli-Q ultrapure water (18.2 MΩ cm^−1^), stirring magnetically for 3 h. Then, the solution was transferred to a three-port flask. Pure nitrogen was used to drive out the dissolved oxygen, and 0.5 mol L^−1^ NaBH_4_ 50 mL was dropped into the suspension until the black substance was formed in the solution. Then, the solution was continuously stirred for 30 min, filtered and separated, washed with ethanol 3 times, and dried in a vacuum at 0 °C overnight. The hydrated carbon of rice straw loaded with nZVI (RC-nZVI) was prepared.

### 2.3. Characterizations

The external structure and morphology characteristics of the synthesized composites were investigated by field emission scanning electron microscopy (Zeiss Merlin Compact FE-SEM and EDS). Surface elemental compositions and its morphology distribution maps were visualized with the SEM equipped with energy-dispersive X-ray spectroscopy (EDS).

### 2.4. Collection of Chromium-Contaminated Soil Samples

**Figure 2:**
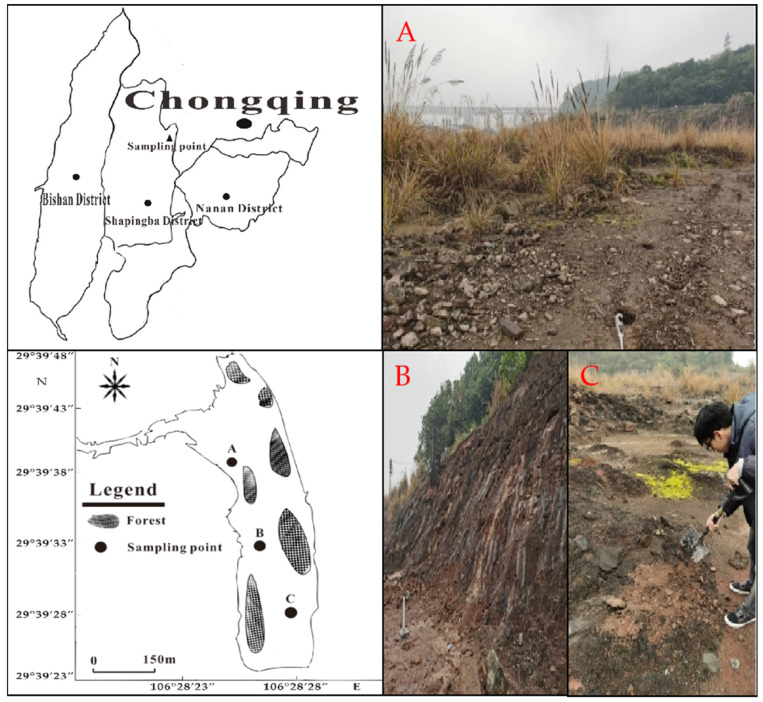
Sampling sites in Chongqing. (**A**) 29°39′40.3″ N, 106°28′24.1″ E, (**B**) 29°39′32.8″ N, 106°28′25.9″ E, (**C**) 29°39′28.4″ N, 106°28′27.6″ E.

The soil samples were collected from a relocated chromium salt plant site (29°39′24″–29°39′48″ N, 106°28′18″–106°28′28″ E) in Chongqing, and the sampling sites are shown in Figure 2. The soil was collected from the surface layer (0–20 cm) under the production workshop and chromium slag pile in the plant area. In each sampling point, the chessboard method is divided into 6 points. A total of 1 kg of soil samples were taken by quartering method and put into sample bags, and a total of 18 kg of soil samples were collected from 18 sampling points. The soil type is mainly yellow-brown soil. The soil samples were naturally air dried to remove plant roots, stones, and other debris, ground through a 3 mm sieve, air dried and sieved, and the samples at each point were mixed well for subsequent use. The physical and chemical properties of soil samples are shown in Table 1.

### 2.5. Analysis Method

#### Cr(VI) Concentration

Content of soil Cr(VI): take 4.0 g of soil samples from the relocation site, put them in a 100 mL beaker, add 50 mL of 0.4 mol L^−1^ KCl solution (v_1_), and stir for 5 min. Transfer the supernatant to a centrifuge tube, centrifuge at 4000 r min^−1^ for 2 min, take a certain amount of supernatant (v_2_) over 0.45 μm filter membrane, and transfer to a 50 mL volumetric flask with a fixed volume (v_3_) to obtain soil extract. The concentration of Cr(VI) (C_1_) was determined by diphenylcarbazide spectrophotometry in an appropriate amount of extractive sample, and the Cr^6+^ content in soil was calculated as C(Cr^6+^ mg kg^−1^) according to the following Equation (1):

(1) $$C\left({\mathrm{Cr}}^{6+}{\;\mathrm{mg}\;\mathrm{kg}}^{-1}\right)=\frac{C_{1}{\;v}_{1}}{w}\times \frac{v_{3}}{v_{2}}$$

where w is the soil sample weight (g), v_1_ is the soil extracting solution volume (mL), v_2_ is the water sampling volume (mL), and v_3_ is the constant volume of sample solution(mL); C(Cr^6+^) denotes Cr(VI) content of soil (mg kg^−1^). C_1_ is the Cr(VI) concentration of the solution (μg mL^−1^). A, B, and C three sites soil sample was mixed equally, and the Cr(VI) content of soil sample was measured as 182.9 mg kg^−1^.

Effect of C/Fe ratio: 0.01 g RC-nZVI was mixed with 4 g 182.9 mg kg^−1^ soil samples while maintaining field moisture capacity (1:1). The solution reaction temperature was set at 25 ± 1 °C, pH = 7, and the reaction time was 60 h. According to the time (0 h, 10 h, 20 h, 30 h, 40 h, 50 h, 60 h), 50 mL of 0.4 mol L^−1^ KCl solution was used to extract Cr(VI). The effect of the mass ratio of C/Fe (1:2, 1:1, 5:4, 5:2, 5:1) on the Cr(VI) reduction rate was investigated.

The effect of initial pH: 0.01 g RC-nZVI was mixed with 4 g 182.9 mg kg^−1^ soil samples while maintaining field moisture capacity (1:1). The reaction temperature was 25 ± 1 °C. Cr(VI) was extracted by 50 mL of 0.4 mol L^−1^ KCl solution, and different initial pHs (2, 4, 6, 8, 10) were adjusted by hydrochloric acid (0.1 mol L^−1^) or sodium hydroxide solution (0.1 mol L^−1^). RC-nZVI was shaken at 120 r min^−1^ for 120 min in a water bath thermostatic oscillator. Samples were taken at different times (0 min, 10 min, 20 min, 30 min, 60 min, 80 min, 100 min, 120 min) and filtered with 0.45 μm filter head. The absorbance was measured to investigate the effect of pH on the reduction of Cr(VI) by RC-nZVI.

The effect of initial temperature: 4 g 182.9 mg kg^−1^ soil sample was added to 0.01 g RC-nZVI while maintaining field moisture capacity (1:1). The initial temperature was set at 20 °C, 30 °C, 40 °C, 50 °C, and 60 °C, respectively. According to the time (0 h, 10 h, 20 h, 30 h, 40 h, 50 h, 60 h), the Cr(VI) was extracted by 50 mL of 0.4 mol L^−1^ KCl solution. The mixture was shaken at 120 r min^−1^ for 120 min in a water bath thermostatic oscillator, and the absorbance was measured by filtering with the 0.45 μm filter head. The effects of initial temperature (20 °C, 30 °C, 40 °C, 50 °C, 60 °C) on the reduction of Cr(VI) by RC-nZVI were investigated.

The effect of initial concentration of Cr(VI): the soil extract was diluted, and the initial concentration of Cr(VI) was 5.1 mg L^−1^, 10.6 mg L^−1^, 15.5 mg L^−1^, and 20.2 mg L^−1^, the dosage of RC-nZV was 0.01 g, the reaction time was 120 min, and the reaction temperature was 25 ± 1 °C, pH = 7. The RC-nZVI was shaken at a speed of 120 r min^−1^ for 120 min in a water bath constant temperature oscillator, and the samples were taken according to the time (0 min, 10 min, 20 min, 30 min, 60 min, 80 min, 100 min, 120 min). The filter head of 0.45 μm was filtered, and the absorbance was measured to investigate the effect of the initial concentration of Cr(VI) on the reduction of Cr(VI) by RC-nZVI.

The above reactions were carried out in a 100 mL beaker, placed in a shaker at 25 °C, with a rotation speed of 120 r. According to the sampling time, 2 mL of the sample was taken with a 5 mL syringe each time, filtered with a 0.45 μm filter head, and diluted 25 times with water in a 50 mL colorimetric tube. Then, the H_2_SO_4_ (aq) (1:1) 0.5 mL, H_3_PO_4_ (aq) (1:1) 0.5 mL, and chromogenic agent 2 mL were added to the colorimetric tube and then stood for 10 min until the color was completely developed.

Cr(VI) concentration in the supernatant was determined by diphenylcarbazide ultraviolet spectrophotometry. The wavelength of the UV spectrophotometer was 540 nm. The supernatant was filtered by the 0.45 μm water system needle filter.

The reduction efficiency of chromium (η, %) was calculated according to the following Equation (2):

(2) $$\eta \;=\frac{C_{0}-{\;C}_{e}}{C_{0}}$$

where C_0_ and C_e_ were Cr(VI) concentration of in solution before and after adsorption, mg L^−1^; η denotes reduction efficiency of Cr, %. Three parallel samples were conducted for each sample, and two blank samples were set as controls.

Organic matter was determined according to NY/T 1121.6-2006 in the soil samples of the relocated chromium salt factory and the titration was performed with ZDJ-5 automatic titrator. Two standard soils and three blanks were used for each batch of digestion.

## 3. Results and Discussion

### 3.1. Characterizations

#### 3.1.1. SEM Images of RC-nZVI Samples

**Figure 3:**
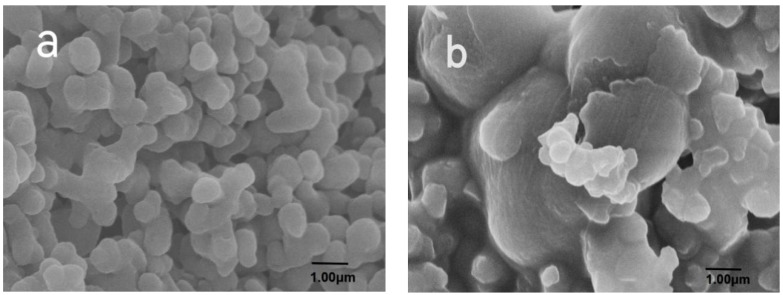
The SEM image of (**a**) blank RC-nZVI and (**b**) RC-nZVI after reaction with Cr(VI) = 5.1 mg L^−1^.

SEM pictures of RC-nZVI before and after the reaction with heavy metal Cr(VI) were illustrated in Figure 3, which showed that the Fe particle presented in spherical shape in uniformly distributed nanometer scale before the reaction with Cr(VI) (Figure 3a). The morphology of samples changed considerably after the reaction with Cr(VI) with bulk components attached on the surface (Figure 3b).

#### 3.1.2. FE-SEM and EDS Analysis of Composite Materials

**Figure 4:**
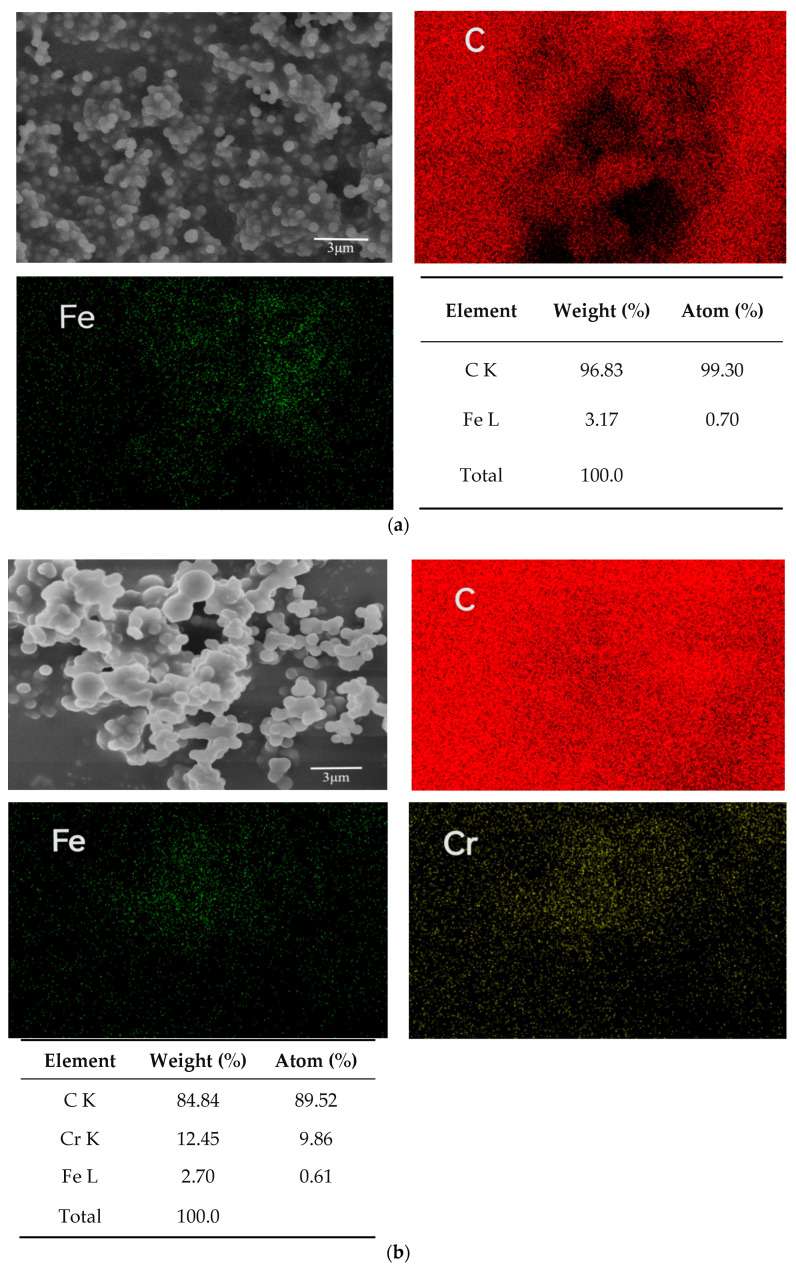
Structure and morphography of RC-nZVI detected with FE-SEM and EDS: (**a**) RC-nZVI; (**b**) RC-nZVI after reaction with Cr(VI) = 5.1 mg L^−1^.

To further reveal the mechanism of Cr(VI) reduction by the synthesized materials, surface characteristics of RC-ZVI before and after reaction with Cr were observed with field emission scanning electron microscopy (FE-SEM and EDS). SEM was detected to explore the element distribution characteristics for the synthesized materials (Figure 4). Results of element mapping indicated that Fe and C elements were evenly distributed on the supporting materials of hydrothermal carbon for RC-nZVI. The chemical composition of the material was analyzed by EDS. The elements before the reaction were Fe and C, and the peak of the Cr element was increased after the reaction. The percentage of Fe in RC-nZVI decreased to 0.61% after the reaction, and the percentage of Cr increased from 0% to 9.86%.

### 3.2. Effect of C/Fe on the Reduction of Soil Cr(VI) by RC-nZVI

**Figure 5:**
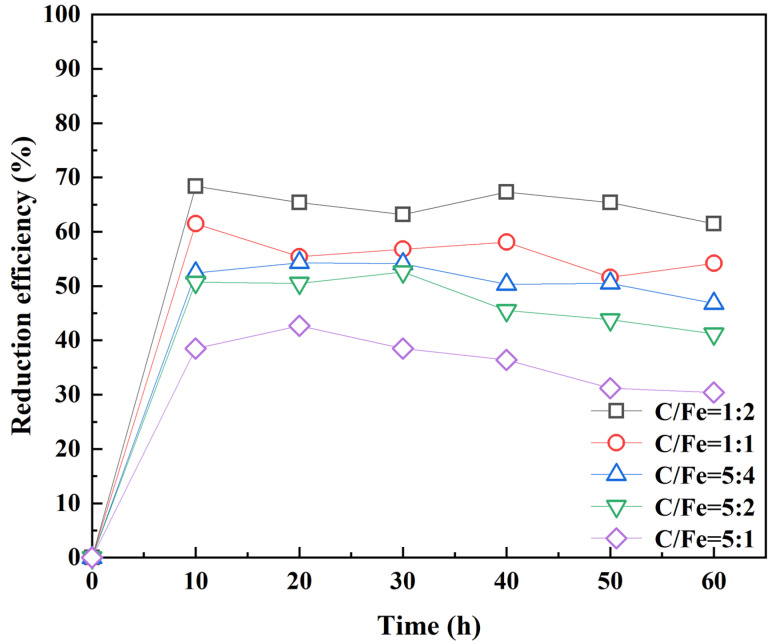
Effect of C/Fe on reducing soil Cr(VI) by RC-nZVI.

As can be seen from Figure 5, the ratio of hydrothermal carbon and nZVI has a more noticeable effect on the Cr(VI) reduction rate. It can be seen from Figure 5 that all soil mixture samples reacted rapidly within the first 10 h, and the Cr(VI) reduction rate reached the peak, and the Cr(VI) reduction rate decreased gradually with time. As the C content increases, Fe^0^ content is constantly decreasing. Therefore, the Cr(VI) reduction rate decreases. It is possible that the reduction of iron may be more critical than the adsorption of carbon.

After 60 h of reaction, the reduction of Cr(VI) decreased to 61.5%, 54.2%, 46.8%, 41.2%, and 30.4% in the different of ratio C/Fe (1:2, 1:1, 5:4, 5:2, and 5:1), respectively. With the reaction time, the rate of Cr(VI) reduction decreased gradually. The reason may be due to the larger mass ratio of hydrothermal carbon to nano zero-valent iron. In the initial stage, the nano zero-valent iron fully reacted, and the Cr–Fe products after the reaction adhered to the surface of the material and formed a passivation layer, which limited the adsorption of hydrothermal carbon [27]. The reason also may be a huge buffer with soil, and organic matter, colloid, and Cr(VI) are continuously adsorbed and resolved. When there is a high ratio of C/Fe and huge content of Cr(VI) in the soil, the reduction of Cr(VI) by Fe^0^ is limited. As the reaction time extends, the reduction rate of Cr(VI) will decrease to some extent. This is consistent with the dangerous effect of Cr(VI) governance in the actual site [28]. Therefore, the relative contribution to the reduction rate of Cr(VI) in the later stage decreased instead.

### 3.3. pH Influence on Cr(VI) Reduction

**Figure 6:**
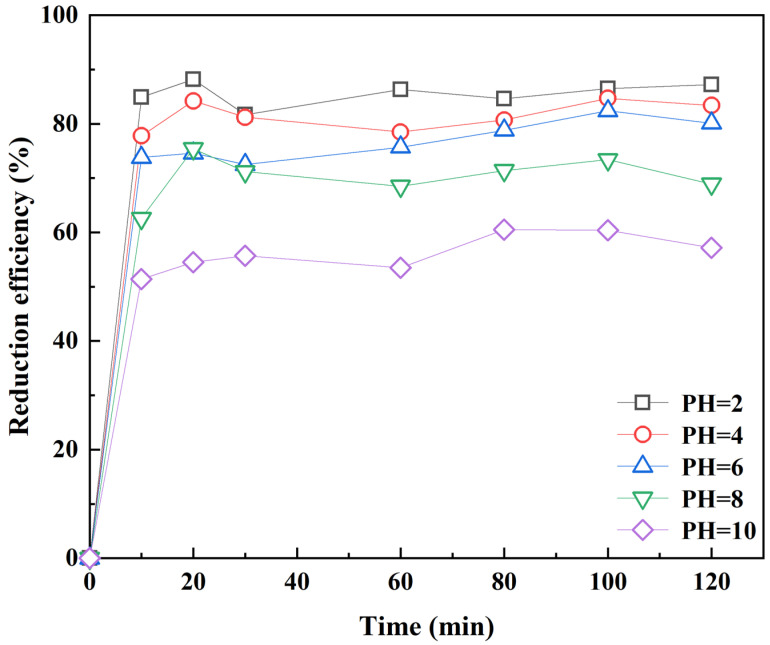
Effect of initial pH on Cr(VI) reduction by RC-nZVI.

The effect of pH on Cr(VI) reduction by RC-nZVI is shown in Figure 6. It can be seen from Figure 6 that the initial pH value of the solution has a significant effect on the reduction efficiency of RC-nZVI.

When pH = 10, the reduction rate of Cr(VI) in the soil was 51.4%. When the pH decreased from 10 to 2, the reduction rate of Cr(VI) by RC-nZVI increased from 51.4% to 84.9%, indicating that acidic conditions were beneficial for the reduction of Cr(VI). The pH has a significant influence on the existing form of Cr in the soil. When pH is less than 4, Cr mainly exists in the form of HCrO^4−^. When the pH is greater than 4, it exists in the form of CrO_4_^2−^ and Cr_2_O_7_^2−^ with the increase of pH. Among them, the adsorption-free energy of HCrO^4-^ is higher than that of CrO_4_^2−^ and Cr_2_O_7_^2−^. Therefore, HCrO^4−^ is more easily adsorbed on the surface of RC-nZVI under acidic conditions, which promotes the adsorption reaction of chromium [29]. At the same time, nano zero-valent iron is positively charged. Hydrothermal carbon has a multi-stage microporous structure and a large specific surface area. Its surface carries negative charge groups of hydroxyl and carboxyl groups, and electrostatically repels negatively charged Cr(VI) [30,31]. On the other hand, reducing Cr(VI) by nZVI provides sufficient H^+^ for Cr(VI) reduction under acidic conditions, which is conducive to the formation of trivalent chromium.

### 3.4. Effect of Temperature on Cr(VI) Reduction by RC-nZVI

**Figure 7:**
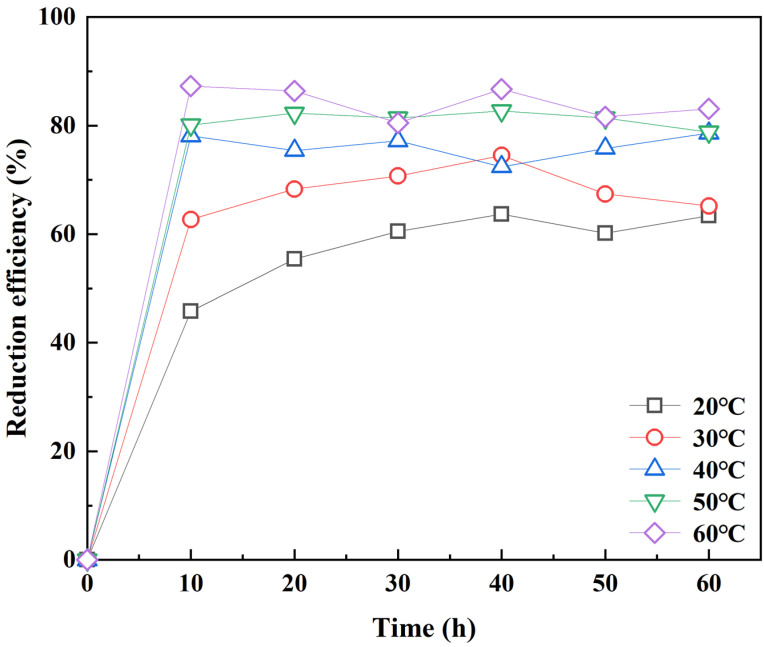
Effect of initial temperature on Cr(VI) reduction by RC-nZVI.

The effect of reaction temperature on the reduction of Cr(VI) by RC-nZVI is shown in Figure 7.

The reduction rates of Cr(VI) were 63.4%, 65.2%, 78.6%, 78.8%, and 83.1% at the reaction temperature conditions of 20 °C, 30 °C, 40 °C, 50 °C, and 60 °C, respectively, during the 60 h reaction time. With the higher temperature, the reduction efficiency of Cr(VI) increases. The movement rate of Cr(VI) in water was accelerated to improve the probability of collision between pollutants and RC-nZVI, and the reduction rate of Cr(VI) was increased [4]. When the temperature increased from 30 °C to 60 °C and the reaction time exceeded 30 h, the reduction rate of Cr(VI) decreased slightly.

### 3.5. Effect of Initial Cr(VI) Concentration on the Reduction Rate and Reaction Kinetics of RC-nZVI

**Figure 8:**
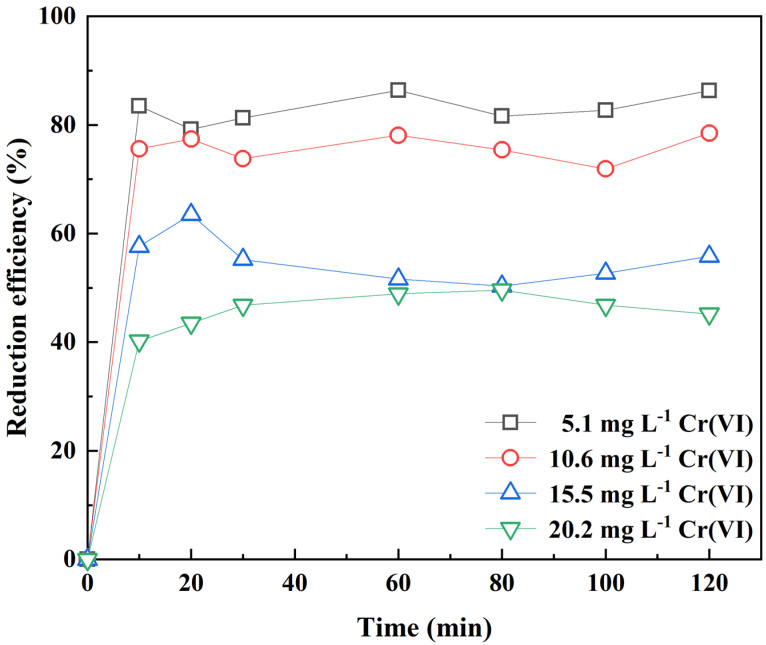
Effect of initial Cr(VI) concentration on the Cr(VI) reduction by RC-nZVI.

The relationship between the initial concentration of Cr(VI) and the Cr(VI) reduction rate by RC-nZVI is shown in Figure 8. In the initial concentration of 5.1–20.2 mg L^−1^ solution, the Cr(VI) reduction rate decreased with increasing initial Cr(VI) concentration. When the initial concentration of Cr(VI) was 5.1 mg L^−1^, the reduction rate was 83% within 90 min of reaction time. In the initial 20 min, the reduction rate of Cr(VI) by RC-nZVI was fast, and Cr(VI) ion in the solution was quickly adsorbed on the nZVI active sites on the material surface. As the reaction continues, the adsorption sites on the material surface gradually become saturated [28]. Under the conditions of C/Fe = 1:2, 60 °C, with pH of 2, the average content of Cr(VI) in soil decreased from 182.9 mg kg^−1^ to 21.6 mg kg^−1^.

The adsorption kinetics process of solid adsorbent for the solute in the solution can be described by pseudo-first-order model, pseudo-second-order model, and intra-particle diffusion model. For the first-order model, the Lagergren first-order rate equation on the basis of solid adsorption is the most common type, and the model formula is given as the following Equation (3):

(3) $$\log\left(q_{e}\;-{\;q}_{t}\right)=\log q_{e}\;-\;\frac{k_{1}t}{2.303}$$

where t means time, in min; q_e_ means equilibrium adsorption capacity, in mg g^−1^; q_t_ means instantaneous adsorption capacity, in mg g^−1^; and k_1_ means the first-order adsorption rate constant, in g mg^−1^ min^−1^.

Pseudo-second-order model is based on the assumption that the adsorption rate is dominated by the mechanism of chemical adsorption, meaning that the adsorption process involves the electron sharing and transfer between adsorbent and adsorbate. The mathematic model is given as the following Equation (4):

(4) $$\frac{t}{q_{t}}=\frac{1}{k_{2}q_{e}^{2}}+\frac{t}{q_{e}}$$

where k_2_ denotes the second-order adsorption constant, in g mg^−1^ min^−1^.

It can be seen from Table 2 that the adsorption kinetics fitting results were significantly affected by the initial Cr(VI) concentration. The 5.1–20.2 mg L^−1^ of Cr(VI) solution was treated with RC-nZVI. The pseudo-second-order kinetic equation fitted well with the average R^2^ of 0.994, the adsorption rate constant of the average adsorption rate constant k_2_ of 0.579 g mg^−1^ min^−1^, and the equilibrium adsorption amount q_e_ of 9.12 mg g^−1^. Both R^2^ and equilibrium adsorption amounts were near to the experimental data. Thus, it can be concluded that the process of Cr(VI) reduction in water by RC-nZVI can be described by the pseudo-second-order kinetic equation. Therefore, the reduction of Cr(VI) by RC-nZVI was mainly dominated by the chemical adsorption process [14].

The reduction rate was accelerated by increasing the concentration of Cr(VI). Moreover, when the Cr(VI) concentration increased to a certain limit, the reduced Cr(VI) separated from the solution in the form of hydroxide co-precipitation, which covered the active sites on the nano zero-valent iron surface and formed a passivation layer on its surface, slowing down the reaction rate [32]. Therefore, the reduction of Cr(VI) by RC-nZVI is mainly controlled by the chemisorption process.

## 4. Conclusions

In this study, functional hydrothermal carbon (RC-nZVI) was synthesized by loading nZVI on hydrothermal carbon. The material (RC-nZVI) achieved good results in removing the Cr(VI) of relocated factory area soil. Under the conditions of C/Fe = 1:2, 60 °C, with pH of 2, the average content of Cr(VI) in soil decreased from 182.9 mg kg^−1^ to 21.6 mg kg^−1^. SEM images showed that nZVI uniformly distributed on the surface of hydrothermal carbon and effectively reduced iron agglomeration. The adsorption kinetics fit well with the pseudo-second-order kinetic model. The velocity constant showed that the reduction rate decreased with increasing initial Cr(VI) concentration. The reduction of Cr(VI) by RC-nZVI was mainly dominated by the chemical adsorption process.

## Figures and Tables

**Table 1:** Basic physical and chemical properties of soil samples.

Soil Sample	PH	CEC (cmol kg^−1^)	Organic Matter (g kg^−1^)	Cr(VI) (mg kg^−1^)
A	7.89 ± 0.01	17.41 ± 0.03	1.25 ± 0.06	176.5 ± 1.7
B	7.82 ± 0.02	14.32 ± 0.07	1.36 ± 0.10	203.4 ± 3.2
C	7.76 ± 0.01	16.89 ± 0.04	1.33 ± 0.05	168.8 ± 2.5

**Table 2:** Fitting parameter of the pseudo-first-order model and pseudo-second-order model for Cr(VI) reduction by RC-nZVI.

Initial Concentration mg L^−1^	Pseudo-First-Order Model			Pseudo-Second-Order Model			q_e_(exp) (mg g^−1^)
	k_1_ (min^−1^)	q_e_(cal) (mg g^−1^)	R^2^	k_2_ (g mg^−1^ min^−1^)	q_e_(cal) (mg g^−1^)	R^2^	
5.1	0.064	2.5116	0.9805	0.813	7.8823	0.9906	8.40
10.6	0.025	2.7702	0.8152	0.706	9.1046	0.9986	11.46
15.5	0.0093	2.7229	0.5327	0.559	8.5397	0.9953	14.68
20.2	0.0072	2.8523	0.6032	0.236	10.9651	0.9928	15.54

## Data Availability

The data presented in this study are available upon request from the corresponding author.
